# Evaluation of Dry Eye Parameters in Patients with Papillary Thyroid Carcinoma

**DOI:** 10.3390/jcm15093336

**Published:** 2026-04-27

**Authors:** Müge Keskin, Belma Özlem Tural Balsak, Neslihan Bayraktar, Çağlar Keskin, Fatma Dilek Dellal Kahramanca, Rıza Gökhan Baykal, Didem Özdemir, Oya Topaloğlu, Reyhan Ersoy, Bekir Çakır

**Affiliations:** 1 Department of Endocrinology and Metabolism, Ankara Bilkent City Hospital, Health Sciences University, Ankara 06800, Turkey; belmabalsak@gmail.com (B.Ö.T.B.); caglaron@hotmail.com (Ç.K.); drdellal@yahoo.com (F.D.D.K.); 2 Department of Ophthalmology, Ankara Bilkent City Hospital, Ankara 06800, Turkey; dr.neslibayraktar@gmail.com; 3 Department of Endocrinology and Metabolism, Ankara Bilkent City Hospital, Ankara 06800, Turkey; gokhanbaykal89@yahoo.com; 4 Department of Endocrinology and Metabolism, Faculty of Medicine, Ankara Yildirim Beyazit University, Ankara 06800, Turkey; sendidem2002@yahoo.com (D.Ö.); oyasude@gmail.com (O.T.); reyhanersoy@yahoo.com.tr (R.E.); drcakir@yahoo.com (B.Ç.)

**Keywords:** papillary thyroid cancer, radioactive iodine treatment, dry eye disease, TSH suppression, meibomian gland dysfunction

## Abstract

**Background/Objectives**: Dry eye disease (DED) is now widely considered to be the most prevalent ocular surface-related disease and has attracted increasing clinical attention in recent years. DED is well-studied in thyroid orbitopathy, but scarce data are available in patients with papillary thyroid carcinoma (PTC). **Methods**: We analyzed 29 PTC cases with radioactive iodine (RAI) treatment (Group 1), 22 PTC cases without RAI (Group 2), and 26 normal control individuals (Group 3). All participants were evaluated with the Ocular Surface Disease Index (OSDI), meibomian gland secretion quality, and lid margin grading. Non-contact meibography and non-invasive tear breakup time measurements were completed using the Sirius Scheimpflug camera. Thyrotropin (TSH), free thyroxine, and free triiodothyronine were measured. **Results**: TSH values were significantly lower in both patient groups than in controls (*p* < 0.001). The proportion of participants with a lower meibomian gland secretion quality score ≥ 1 was 34.5% in Group 1, 22.7% in Group 2, and 7.1% in Group 3 (*p* = 0.002). There was a higher proportion of a meibomian gland atrophy score ≥ 1 in Groups 1 and 2 than in controls (*p* = 0.007). No differences were found between groups in lower lid margin score, Oxford values, upper meibomian gland expressibility or upper meibography scores (*p* = 0.485, *p* = 0.064, *p* = 0.256, *p* = 0.069). OSDI scores were higher in both PTC groups than in controls (6.25 and 8.12 vs. 2.52), with borderline overall significance (*p* = 0.050) and higher pairwise scores versus controls (both *p* = 0.034). **Conclusions**: Meibomian gland dysfunction was observed in PTC patients regardless of RAI treatment, suggesting that TSH suppression itself may contribute to the development of ocular surface changes.

## 1. Introduction

The most common type of thyroid cancer is papillary thyroid carcinoma which represents about 90% of all cases. There has been an increasing incidence of differentiated thyroid cancer (DTC) during the past 4 decades in all age groups of adults, but this increase has been accompanied by better survival due to combined surgery and treatment with radioactive iodine (RAI) [1]. As reported by the Surveillance, Epidemiology, and End Results (SEER) of National cancer institute, the 5-year relative survival of thyroid cancer was 98.4% between 2014–2020 [2]. Dry eye disease (DED) is now recognized as the most common ocular surface disorder worldwide. In the last 30 years, the awareness of DED has increased significantly worldwide. In 2007, the TFOS DEWS report was the first to define dry eye as a disease, and the TFOS DEWS II report revised the definition. It is defined as a multifactorial ocular surface disease that is linked to ocular symptoms and is typified by tear film instability. The etiology of the disease includes ocular surface inflammation and damage, tear film instability and hyperosmolarity, and neurosensory abnormalities [3]. DED leads to visual impairments and discomfort [4]. The pathophysiology of DED may involve dysfunction of the lacrimal functional unit and/or the meibomian glands. These sebaceous holocrine glands, located within the upper and lower eyelids, contribute critically to tear-film homeostasis by secreting lipids that form the outer tear-film layer, thereby reducing evaporation and stabilizing the ocular surface. In addition to lipids, the meibomian glands also contribute biologically active factors, including proteins such as ectodysplasin A, which may support ocular surface integrity and epithelial homeostasis [5]. DED is a common symptom among patients with thyroid orbitopathy due to Graves’ disease (GD), with a rate of 65.2% [6].

In the present study, we aimed to evaluate the dry eye parameters of PTC patients who received or did not receive RAI treatment. We hypothesized that DED parameters would be more adversely affected in PTC patients, especially those exposed to RAI therapy or under long-term TSH suppression, compared to healthy controls. This study will determine the incidence of DED and its causes (RAI or L-thyroxine suppression) in a specific group of patients with PTC. Our findings may help raise clinical awareness and improve the management of DED in patients with PTC.

## 2. Materials and Methods

### 2.1. Study Design

Fifty-one patients (aged ≥ 18 years) with papillary thyroid carcinoma (PTC) who had undergone thyroidectomy and attended our clinic were recruited for this single-center, observational cross-sectional study with retrospective clinical data review and prospective ophthalmological assessment (June 2023–July 2024). Among them, 29 patients who had received RAI therapy were allocated to Group 1 and 22 patients without RAI to Group 2. An additional 26 healthy individuals with no history of thyroid cancer were enrolled as controls (Group 3). Controls were recruited from hospital staff and companions and were frequency-matched on age and sex where feasible; individuals with thyroid disease history or abnormal screening TSH were excluded. Primary endpoints were prespecified as lower-lid meibomian gland expressibility/meibography and non-invasive tear break-up time (NITBUT); upper-lid measurements were collected as supportive/exploratory outcomes to characterize within-subject concordance, not to test a lid-specific hypothesis.

### 2.2. Sample Size and Eligibility

The sample size was based on consecutive eligibility rather than a priori power calculation; accordingly, the study is hypothesis-generating. To aid interpretability, we report effect sizes and 95% confidence intervals and avoid inferring absence of effect from non-significant results. Although no formal power analysis was performed due to the retrospective and observational design, the total number of participants was considered sufficient to identify clinically meaningful differences in dry eye parameters among the three groups. The inclusion of a healthy control group improves interpretability and allows comparative assessment of the impacts of RAI exposure and thyroid hormone suppression on ocular surface health. Exclusion criteria were diabetes; rheumatologic diseases; ocular conditions (keratoconus, glaucoma, chronic allergic conjunctivitis, corneal scarring, and pterygium); history of contact lens wear; and prior ocular surgery. In addition, participants with clinically overt ocular surface disease on history or ophthalmologic examination were not included. Patients who had received RAI at least three years before assessment were eligible for the RAI-treated group. For RAI-treated patients, we recorded latency since last RAI (years) and, where available, cumulative administered activity (mCi) from medical records to explore dose–response or time-since-exposure relationships with ocular surface parameters.

### 2.3. Hormone Assays and Ocular Assessments

Morning serum thyrotropin (TSH), free thyroxine (fT4), and free triiodothyronine (fT3) were measured using a chemiluminescent immunoassay (CLIA) with a Siemens Atellica analyzer. Dry eye symptoms were assessed with the 12-item Ocular Surface Disease Index (OSDI) questionnaire prior to examination [7]. Meibomian gland secretion quality, Oxford staining score, and lid margin score were evaluated by slit-lamp; NITBUT and meibography were measured using the Sirius device (CSO, Costruzione Strumenti Oftalmici, Florence, Italy). Two masked ophthalmologists, following a joint calibration session, performed all examinations. For patient-level analyses, measurements from both eyes were averaged to avoid inter-eye dependence; upper–lower lid indices were additionally summarized to assess within-subject patterns. All examinations were conducted according to a standardized protocol in order to minimize inter-observer variability.

### 2.4. Grading Scales

Lid margin scoring was as follows: 0: normal; 1: irregularity of the lid margin; 2: vascular congestion of the lid margin; 3: obstruction of the lid margin gland orifices; 4: displacement of the mucocutaneous junction. The quality of meibomian gland secretion was graded as follows: 0: clear meibomian secretion with light compression; 1: cloudy meibomian secretion with light compression; 2: cloudy meibomian secretion with moderate compression; 3: no meibomian gland secretion despite strong compression. The meibography score (meibomian gland atrophy score) was graded on a scale from 0 to 4 in the upper and lower lids as follows: 0: no atrophy; 1: atrophy area < 25%; 2: atrophy area 26–50%; 3: atrophy area 51–75%; 4: atrophy area > 75% [8].

### 2.5. Statistical Analysis

Continuous variables were summarized as mean ± standard deviation (SD) if normally distributed, or median (min–max) otherwise; categorical variables are presented as numbers (%). Alongside *p*-values, we report effect sizes (η^2^ for ANOVA; r for Mann–Whitney) and 95% confidence intervals where appropriate. Normality was assessed using the Kolmogorov–Smirnov test. Group comparisons used one-way ANOVA with Bonferroni post hoc tests for normally distributed variables and Kruskal–Wallis tests with Bonferroni-corrected Mann–Whitney U tests for non-normal variables. For patient-level primary analyses, outcomes were computed as the average of both eyes. In a sensitivity analysis, we fitted GEE models to account for within-subject inter-eye correlation for eye-level outcomes. Correlations between TSH/fT4 and ocular outcomes (OSDI, NITBUT, meibography) were examined using Spearman’s ρ; age- and sex-adjusted associations were additionally evaluated using rank-based or robust regression, as appropriate. Smoking status and antihypertensive medication use were also explored in sensitivity analyses where available. Categorical variables were compared using the chi-square (or Fisher’s exact) test. All analyses were conducted using IBM SPSS version 26.0 (IBM Corporation, Armonk, NY, USA). A *p*-value of <0.05 was considered statistically significant. Bonferroni adjustment was performed to account for the test of multiple hypotheses in pairwise comparisons in non-parametric analysis, with the significance level at *p* < 0.017 (0.05/3), for the three prespecified pairwise comparisons of primary endpoints.

## 3. Results

Mean age and sex ratio were comparable between the groups (*p* = 0.115 and *p* = 0.852, respectively). When TSH, fT3, and fT4 levels were compared between the groups, TSH values were significantly lower in the RAI-positive (*n* = 29) and RAI-negative (*n* = 22) patient groups compared to the control group (*n* = 26) (*p* < 0.001 for each), and fT4 values were significantly higher in the RAI-positive and negative patient groups compared to the control group (*p* < 0.001 and *p* = 0.003, respectively). OSDI scores were 6.25 in Group 1, 8.12 in Group 2, and 2.52 in the control group. The overall three-group comparison was of borderline significance (*p* = 0.050), while pairwise comparisons showed higher OSDI scores in both Group 1 and Group 2 than in controls (both *p* = 0.034) (Table 1).

There was no significant difference in the proportion of patients with normal mean noninvasive tear breakup time (≥17) (*p* = 0.172) or the proportion of patients with normal first noninvasive tear breakup time (≥17) (*p* = 0.182) between Group 1, Group 2, and the control group. Lower meibography values were significantly higher in the RAI-positive and negative patient groups compared to the control group (*p* = 0.010 and *p* = 0.019, respectively). No significant differences were found between the groups in terms of upper or lower lid margin score, Oxford score, upper or lower meibomian gland secretion quality, or upper meibography values (Table 2).

The proportion of those with an upper lid margin score of ≥1 was 19.2% in the control group, 35.1% in the positive group, and 43.2% in the negative group (*p* = 0.041). The proportion of those with lower meibomian gland secretion quality of ≥1 was 7.7% in the control group, 34.5% in the positive group, and 22.7% in the negative group (*p* = 0.002). The proportion of patients with a lower meibography grade of ≥1 was 26.2% in the control group, 61.1% in the positive group, and 52.5% in the negative group (*p* = 0.004). Lower lid margin score, Oxford score, upper meibomian gland secretion quality, or upper meibography grades were not different between the groups (Table 3).

## 4. Discussion

Meibomian gland dysfunction (MGD) is the most common cause of DED [9]. In the current study, meibomian gland dysfunction was shown in PTC patients who received or did not receive RAI treatment. In our cohort, the absence of a clear difference between the RAI-treated and untreated groups suggests that long-term thyroid hormone suppression, rather than prior RAI exposure, may be the more persistent determinant of ocular surface alterations. Thyroid hormone suppression therapy (THST) is a strategy to suppress TSH levels in patients with DTC to achieve better clinical outcomes and reduce recurrence and mortality [10,11]. Iatrogenic hyperthyroidism following THST can be associated with osteoporosis, fractures, and cardiovascular disorders including atrial fibrillation [12].

Recently, attempts have been made to understand the contribution of apoptosis in the pathophysiology of DED. Reactive oxygen species (ROS) initiate lipid peroxidation and induction of proinflammatory mediators in the tear fluid and production of inflammatory cytokines that result in damage to the corneal and conjunctival cells and cell death secondary to apoptosis [13]. ROS generation and lipid peroxidation are enhanced during hyperthyroidism.

In a study conducted with patients with thyroid orbitopathy and Graves’ disease (GD), meibomian gland dysfunction (MGD) was more severe and lipid layer thickness was greater in those with active ophthalmopathy [6]. TSH concentrations were suppressed to 0.41 ± 0.11 mU/L in the RAI-positive group, 0.53 ± 0.16 mU/L in the non-RAI group, and within the euthyroid range (1.54 ± 0.11 mU/L) in the control group in our study. Potential mechanisms of thyroid-associated ophthalmopathy (TAO) include inflammatory responses involving the lacrimal gland and a decreased/incomplete blink, and meibomian gland dropout, all of which may affect the volume and composition of the tear film [14].

Zhao et al. showed that GD patients with higher TRAb and fT3 at an early stage had a higher probability of developing Graves’ orbitopathy (GO). Their study reveals that the symptoms appear and increase as the level of suppression increases. In our study, we found no difference between the groups in terms of fT3 levels; on the other hand, fT4 levels were significantly higher in both the RAI-positive and RAI-negative groups of patients with PTC [15]. MGD, known to be an important cause of dry eye in patients with thyroid eye disease (TED), causes high evaporation of the tear film and participates in the pathogenesis of DED. It has also been found that lacrimal glands are enlarged in hyperthyroidism patients with TED [16]. Taken together, these findings suggest that THST in patients with PTC may contribute to the development of DED. In this context, the observed ocular surface changes are more plausibly related to the hormonal milieu created by long-term TSH suppression, particularly relative hyperthyroxinemia, than to the presence of thyroid carcinoma itself.

RAI treatment has emerged as a cornerstone of DTC treatment for more than 70 years, with dosing optimization continuing to be an essential consideration in treatment course [17,18]. Although efficacious and relatively safe, RAI is not without complications, most notably those pertaining to the nasolacrimal apparatus. Reported complications include nasolacrimal duct stenosis, sialadenitis, epiphora, and even less commonly, secondary primary malignancies and infertility, all believed to be related to cumulative RAI exposure [19,20,21]. Notably, previous studies have suggested that changes in tear dynamics and ocular surface health may arise in the early post-treatment period following RAI administration.

However, our study did not show a significant difference in dry eye parameters between RAI-treated and untreated groups. The fact that the RAI-treated group in the study was selected from patients who had received it at least 3 years previously may have led to this result. The extended duration since exposure may have diminished or eliminated early inflammatory effects on the ocular surface. Studies indicate that although RAI may cause short-term nasolacrimal toxicity, its long-term effects on the ocular surface seem minimal or reversible [21]. Because RAI is a systemic therapy, a true biological difference between upper- and lower-lid meibomian gland function would not be expected a priori. In our study, lower-lid indices were prespecified as primary endpoints because they are more reliably assessed at the slit-lamp, whereas upper-lid parameters were collected as exploratory outcomes. Given the modest sample size and the greater measurement variability of upper-lid measures, any apparent upper–lower discrepancy is more likely to reflect sampling variability than a true lid-specific effect of RAI. The predominance of lower-lid abnormalities in our study may also reflect anatomical and functional differences between the upper and lower meibomian glands. Although the upper lid contains a greater number and total area of meibomian glands, lower-lid glands are generally easier to visualize and express clinically, which may improve the detectability of mild dysfunction. In addition, local factors such as blink mechanics, lid-margin exposure, and tear pooling may differentially influence lower-lid assessments. Therefore, the stronger lower-lid signal observed here may represent a combination of true physiological susceptibility and greater clinical detectability, rather than a selective biological effect of TSH suppression on the lower lid alone.

The mean age of the patients was 45.37 ± 1.86 years in the RAI-positive group and 45.72 ± 2.51 years in the RAI-negative group. Consistent with SEER data from the United States, thyroid cancer is most frequently diagnosed in adulthood and middle age, particularly between 45 and 64 years of age [8]. The prevalence of thyroid cancer rises with advancing age from adolescence to middle age. These are the times when people are at their most active, both in business and in daily life. Dry eye is a bothersome condition that can affect activities of daily living, work productivity, and quality of life. Due to the growing use of digital tools in daily life, the effects of this condition may be more problematic for those it affects. Given the risk profile, higher clinical suspicion for dry eye disease is warranted among patients with thyroid cancer. If early diagnosis and management are achieved, the patient’s comfort and quality of life may be improved.

This study has several limitations. The sample size was relatively small, which may have limited statistical power and increased the risk of type II error, particularly for parameters showing only borderline or numerical trends. No formal power analysis was conducted, but the number of subjects was considered sufficient to identify clinically significant variations in dry eye across the three groups. The inclusion of a control group further supported the comparative evaluation of ocular surface alterations. Nevertheless, future studies with larger, multi-center cohorts and prospective designs are warranted to validate and extend these findings. In addition, formal inter-observer reproducibility metrics were not calculated, although all examinations were performed by two masked ophthalmologists using a standardized and calibrated protocol. Furthermore, a prior clinical diagnosis of dry eye disease was not used as a formal exclusion criterion, which may have introduced background ocular surface variability despite the exclusion of overt ocular pathology. Secondly, instead of the Schirmer test, we evaluated noninvasive tear breakup time using the Sirius device. The Schirmer test, traditionally used to assess aqueous tear production, has several limitations. It is invasive, it may induce reflex tearing due to mechanical stimulation, and results may vary depending on whether anesthesia is applied. Moreover, its clinical reliability and repeatability have been questioned. Noninvasive diagnostic tools are recommended by the TFOS DEWS II guidelines due to their superior patient comfort and standardized performance in dry eye assessment [22]. More recently, the TFOS DEWS III reports have further refined the contemporary framework for dry eye disease by updating diagnostic methodology and management concepts in light of evidence published since DEWS II [23]. Our use of non-invasive tear film assessment is also consistent with this more recent approach, which emphasizes standardized and reproducible ocular surface evaluation.

## 5. Conclusions

In this study, meibomian gland dysfunction (MGD) was observed in patients with papillary thyroid carcinoma (PTC), independent of their exposure to radioactive iodine (RAI) therapy. Our results indicate that long-term thyroid hormone suppression therapy (THST), rather than thyroid carcinoma itself, may be associated with ocular surface alterations. Given that dry eye disease (DED) can have a considerable effect on the quality of life and activities of daily living, clinicians must remain vigilant in monitoring for DED symptoms in this group of patients. Periodic ophthalmologic evaluation should be considered, especially in patients undergoing life-long TSH suppression, to diagnose and treat ocular surface complications early.

## Figures and Tables

**Table 1:** Comparison of age, sex, and thyrotrophin, free thyroxine, and free triiodothyronine levels between the groups.

	Group 1 RAI-Positive (*n* = 29)	Group 2 RAI-Negative (*n* = 22)	Group 3 Control (*n* = 26)	*p*
Age (mean ± SE)	45.37 ± 1.86	45.72 ± 2.51	40.30 ± 1.78	0.115 *
Sex (*n*, %)				
Female	26 (89.7)	19 (86.4)	22 (84.6)	0.852 **
Male	3 (10.3)	3 (13.6)	4 (15.4)	
TSH (mU/L)	0.41 ± 0.11	0.53 ± 0.16	1.54 ± 0.11	**<0.001** *
fT3 (ng/L)	3.27 ± 0.07	3.48 ± 0.09	3.48 ± 0.06	0.077 *
fT4 (ng/dL)	1.38 ± 0.03	1.50 ± 0.07	1.16 ± 0.02	**<0.001** *
OSDI	6.25 (0–55.55)	8.12 (0–78.57)	2.52 (0–38.88)	0.050 ***

* One-way ANOVA (Post hoc: Bonferroni), ** Chi-squared test, *** Kruskal–Wallis test (Post hoc: Bonferroni-corrected Mann–Whitney U test), SE: Standard Error, RAI: radioactive iodine, TSH: thyrotrophin, fT3: free triiodothyronine, fT4: free thyroxine, OSDI: Ocular surface disease index score.

**Table 2:** Comparison of eye measurements between the groups.

	Group 1 RAI-Positive	Group 2 RAI-Negative	Group 3 Control	*p*
Mean noninvasive tear breakup time (*n*, %)				
>17 (normal)	16 (30.8)	12 (27.3)	23 (44.2)	0.172 *
<17 (pathological/abnormal)	36 (69.2)	32 (72.7)	29 (55.8)	
First mean noninvasive tear breakup time (*n*, %)				
>17 (normal)	16 (31.4)	12 (27.3)	23 (44.2)	0.182 *
<17 (pathological/abnormal)	35 (68.6)	32 (72.7)	29 (55.8)	
Upper lid margin score	0.71 ± 0.14	0.86 ± 0.15	0.38 ± 0.11	0.054 *
Lower lid margin score	0.63 ± 0.13	0.52 ± 0.13	0.36 ± 0.10	0.298 *
Oxford score	0.11 ± 0.04	0.14 ± 0.05	0.01 ± 0.01	0.082 *
Upper meibomian gland secretion quality	0.56 ± 0.11	0.36 ± 0.10	0.36 ± 0.10	0.294 *
Lower meibomian gland secretion quality	0.44 ± 0.08	0.31 ± 0.09	0.15 ± 0.08	0.060 *
Upper meibography grade	0.92 ± 0.13	0.75 ± 0.15	0.88 ± 0.11	0.650 *
Lower meibography grade	0.92 ± 0.13	0.92 ± 0.16	0.35 ± 0.10	**0.005** *

* One-way ANOVA (Post hoc: Bonferroni). (Ratios are calculated according to the total number of eyes.) RAI: radioactive iodine.

**Table 3:** Categorical comparison of eye measurements between the groups.

	Group 1 RAI-Positive	Group 2 RAI-Negative	Group 3 Control	*p*
Upper lid margin score (*n*, %)				
0	37 (64.9)	25 (56.8)	42 (80.8)	**0.041** *
≥1	20 (35.1)	19 (43.2)	10 (19.2)	
Lower lid margin score (*n*, %)				
0	40 (69.0)	32 (72.7)	41 (78.8)	0.554 *
≥1	18 (31.0)	12 (27.3)	11 (21.2)	
Oxford score (*n*, %)				
0	48 (88.9)	36 (85.7)	51 (98.1)	0.082 *
≥1	6 (11.1)	6 (14.3)	1 (1.9)	
Upper meibomian gland secretion quality (*n*, %)				
0	37 (63.8)	34 (77.3)	39 (75.0)	0.290 *
≥1	22 (36.2)	10 (22.7)	13 (25.0)	
Lower meibomian gland secretion quality (*n*, %)				
0	38 (65.5)	34 (77.3)	48 (92.3)	**0.003** *
≥1	20 (34.5)	10 (22.7)	4 (7.7)	
Upper meibography grade (*n*, %)				
0	20 (37.0)	20 (50.0)	15 (35.7)	0.070 *
≥1	34 (63.0)	20 (50.0)	27 (64.3)	
Lower meibography grade (*n*, %)				
0	21 (38.9)	19 (47.5)	31 (73.8)	**0.004** *
≥1	33 (61.1)	21 (52.5)	11 (26.2)	

* Chi-squared test. (Ratios are calculated according to the total number of eyes.) RAI: radioactive iodine.

## Data Availability

The data supporting the findings of this study are available from the corresponding author upon reasonable request.
